# Regulation and Function of TRPM7 in Human Endothelial Cells: TRPM7 as a Potential Novel Regulator of Endothelial Function

**DOI:** 10.1371/journal.pone.0059891

**Published:** 2013-03-22

**Authors:** Erika Baldoli, Sara Castiglioni, Jeanette A. M. Maier

**Affiliations:** Dipartimento di Scienze Biomediche e Cliniche L. Sacco, Università di Milano, Milano, Italy; Biological Research Centre of the Hungarian Academy of Sciences, Hungary

## Abstract

TRPM7, a cation channel of the transient receptor potential channel family, has been identified as a ubiquitous magnesium transporter. We here show that TRPM7 is expressed in endothelial cells isolated from the umbilical vein (HUVEC), widely used as a model of macrovascular endothelium. Quiescence and senescence do not modulate TRPM7 amounts, whereas oxidative stress generated by the addition of hydrogen peroxide increases TRPM7 levels. Moreover, high extracellular magnesium decreases the levels of TRPM7 by activating calpains, while low extracellular magnesium, known to promote endothelial dysfunction, stimulates TRPM7 accumulation partly through the action of free radicals. Indeed, the antioxidant trolox prevents TRPM7 increase by low magnesium. We also demonstrate the unique behaviour of HUVEC in responding to pharmacological and genetic inhibition of TRPM7 with an increase of cell growth and migration. Our results indicate that TRPM7 modulates endothelial behavior and that any condition leading to TRPM7 upregulation might impair endothelial function.

## Introduction

Magnesium (Mg), the second most abundant intracellular cation, plays a major role in regulating endothelial function [1]. Low extracellular Mg retards endothelial cell proliferation and promotes the acquisition of a senescent phenotype, inhibits cell migration, stimulates the adhesion of monocytes, influences the uptake and metabolism of low-density lipoproteins as well as the synthesis of vasoactive molecules such as prostacyclin and nitric oxide [1]. These effects occur, at least in part, through Mg dependent regulation of reactive oxygen species, important signalling molecules involved in modulating endothelial behaviour [2], as well as through Mg-dependent acquisition of an inflammatory phenotype by the endothelial cell [1], [3]. Interestingly, endothelial function is significantly impaired in a model of inherited hypomagnesemia (MgL) in mice [4]. When compared to controls, MgL aortas show reduced amounts of endothelial-nitric oxide synthase (eNOS), which correlate with the decreased levels of plasma nitrate, and increased expression of pro-inflammatory markers such as VCAM and PAI-1 and of the Mg transporter TRPM7 [4]. Although Mg is implicated in many biological processes [5], only recently some light has been shed on the transport mechanisms that regulate its homeostasis. Genetic and electrophysiological studies have identified several Mg entry systems [6], being *TRPM6* and *-7* the first molecularly defined components of the mammalian Mg transport machinery. TRPM6 and -7 show the unique functional duality of being an ion channel and a kinase. TRPM7, which is ubiquitously expressed, was initially thought to play a prominent role in intracellular Mg homeostasis, whereas TRPM6 controls systemic Mg homeostasis by regulating Mg transport in the kidney and in the gut [7]. However, recently TRPM7 has been shown to be critically involved also in systemic Mg regulation [8].

The presence of functional TRPM7 channels in human endothelial cells has been demonstrated [9]. Recently, in human capillary endothelial cells (HMEC) we have shown that the growth-inhibitory effect of low Mg is mediated, at least in part, by the decrease of TRPM7, an event which prevents the influx of Mg necessary for cell proliferation. Accordingly, silencing *TRPM7* mimics the effects of Mg deficiency in these cells [10], thus suggesting TRPM7 as a possible contributor to the regulation of angiogenesis.

In endothelial cells derived from the umbilical vein (HUVEC), two independent reports have shown that siRNAs transiently silencing *TRPM7* stimulate cell proliferation [9], [10], a behaviour which is unique to HUVEC, because in various cell types silencing *TRPM7* induces cell cycle arrest. In addition to the significant elevation of TRPM7 in the vasculature of MgL mice [4], the increase of *TRPM7* transcript in HUVEC exposed to shear stress has been described [11].

To this purpose, it is noteworthy that different types of endothelial cells including HUVEC have very low levels of TRPM7 current which shows no significant increase in response to fluid flow [12]. On the contrary, in smooth muscle cells functional TRPM7 rapidly accumulate at the plasma membrane after exposure to shear stress and this correlates with the increase in TRPM7 current [12].

Although scarce, the data reported until now point to a potential regulatory role for TRPM7 in the maintenance of vascular integrity [13].

Because increasing evidence suggests that TRPM7 might contribute to the pathophysiology of the vasculature in general and of the endothelium in particular, we explored the modulation of the expression of TRPM7 in human endothelial cells and the effects of its inhibition on some aspects of endothelial function.

## Materials and Methods

### Cell Culture

Primary HUVEC isolated from the umbilical vein (American Type Culture Collection) were cultured in M199 containing 10% fetal bovine serum (FBS), 1 mM glutamine, 1 mM penicillin and streptomycin, Endothelial Cell Growth Factor (150 µg/ml), 1 mM sodium pyruvate and heparin (5 units/ml) on 2% gelatin-coated dishes [3]. A Mg free medium (Invitrogen) was utilized to vary the concentrations of Mg by adding MgSO_4_ [3]. Apart from HUVEC rendered senescent by serial passages *in vitro*, HUVEC were used at low population doublings (PD, less than 20). PD were calculated as log_2_ (number of cells at time of subculture/number of cells plated). We define senescent cells as the culture that do not increase the cell number and remain subconfluent for 2 weeks. We confirmed the senescent phenotype with senescence-associated-beta galactosidase activity assay as described [14]. Quiescence was reached by culturing HUVEC for 48 h in starvation medium (M199 containing 1.0% FBS) or by contact inhibition. In some experiments, HUVEC were starved for 48 h to arrest the cell cycle and then exposed to complete growth medium for various times to stimulate the re-entry in the cell cycle [15].

In some experiments HUVEC were treated with H_2_O_2_ (100 µM) for 30 min and then part of the samples immediately lyzed while the remaining was maintained in culture for 24 additional h before lysis. In other experiments HUVEC were treated with trolox (10 µM) [3], MG132 (5 µM), bafilomycin (100 nM) or calpeptin (5 and 10 µg/ml) (Sigma Aldrich).

To silence *TRPM7*, we utilized the pGIPZ shRNAmir (0.2 µg/cm^2^) (Open Biosystems). The construct was transfected into 3×10^5^ HUVEC using Arrest-in Transfection Reagent (Open Biosystems). Transfection with Non-Silencing (scrambled) pGIPZ shRNAmir was used as control.

For proliferation assays, the cells were seeded at low density in growth medium. At the end of the experiments, the cells were trypsinized, stained with a trypan blue solution (0.4%) and the viable cells were counted using a Burker chamber. In some experiments the cells were treated with 2-aminoethoxydiphenyl borate (2-APB, 50 µM) or Co(III)-hexaammine (250 µM) (Sigma Aldrich).

Migration was determined using an *in vitro* model of wound repair as previously described [10]. After wounding, the monolayer was washed and incubated for 10 additional h. The wound area was calculated by ImageJ software and expressed using an arbitrary value scale. All the experiments were performed in triplicate at least three times.

### RT-PCR

Cells were lyzed in 1 ml Trizol and RNA was purified. RNA was quantified using Nanodrop ND-1000 spectrophotometer and electrophorezed on a 1% agarose gel containing 2.2 M formaldehyde before reverse transcription. Using the Transcriptor first-strand cDNA synthesis kit (Roche Diagnostics), cDNA was synthesized from 2 µg of total RNA using oligo-dT and 5 units of reverse transcriptase at 50°C for 60 min, followed by heating to 85°C for 5 min. PCR amplification was carried out using 1/50 of the final RT reaction. Each *TRPM7* amplification cycle consisted of 1 min at 95°C, 45 sec at 54°C and 1 min at 72°C using 400 nmol/l of each primer in a final volume of 25 µl. The reaction was stopped after 35 cycles. One fifth of the reaction mix was separated on a 2% agarose gel. The sequences of the *TRPM7* primers are the following: sense 5′-CTTATGAAGAGGCAGGTCATGG-3′ and antisense 5′-CATCTTGTCTGAAGGACTG-3′ (size of the amplified fragment: 213 bp). RT-PCR with specific primers for actin was performed to normalize (sense 5′-GCATGGAGTCCTGTCGCATCC-3′; antisense 5′-GCGGCCAGGATG GAGCCGC-3′; size of the amplified fragment: 238 bp).

### Western Blot

HUVEC were lyzed in lysis buffer (50 mM TrisHCl pH 8.0, 150 mM NaCl, 1 mM EDTA, 1% NP-40). Protein concentration was determined using the Bradford protein assay (Bio-Rad). Cell extracts (100 µg/lane) were resolved by 8% SDS-PAGE, transferred to nitrocellulose sheets at 100 mA for 16 h, and probed with anti-TRPM7 (Chemicon) and anti-actin (Sigma Aldrich) antibodies. Secondary antibodies were labelled with horseradish peroxidase (GE Healthcare). The SuperSignal chemiluminescence kit (Pierce) was used to detect immunoreactive proteins. All the results shown were reproduced at least three times and a representative Western blot is shown. Densitometry was performed by the ImageJ software on 3–5 different blots.

### NOS Activity

NOS activity was measured in the conditioned media by the Griess method [16]. Briefly, conditioned media were mixed with an equal volume of freshly prepared Griess reagent. The absorbance was measured at 550 nm. The concentrations of NO in the samples were determined using a calibration curve generated with standard NaNO_2_ solutions. The experiments were performed in triplicate and repeated 5 times with similar results. Data are shown as the mean ± standard deviation.

## Results

### Modulation of TRPM7 Expression in Proliferating, Quiescent and Senescent HUVEC

To evaluate whether the expression of TRPM7 is related to cell growth, we compared proliferating vs quiescent HUVEC for their amounts of TRPM7. On culture plates, HUVEC grow until they form a perfect monolayer. At this stage, cells stop growing and become quiescent. This pattern can be shown by the arrest of thymidine incorporation [15]. We first evaluated TRPM7 levels in cells rendered quiescent by growth factor withdrawal or by contact inhibition.

**Figure 1:**
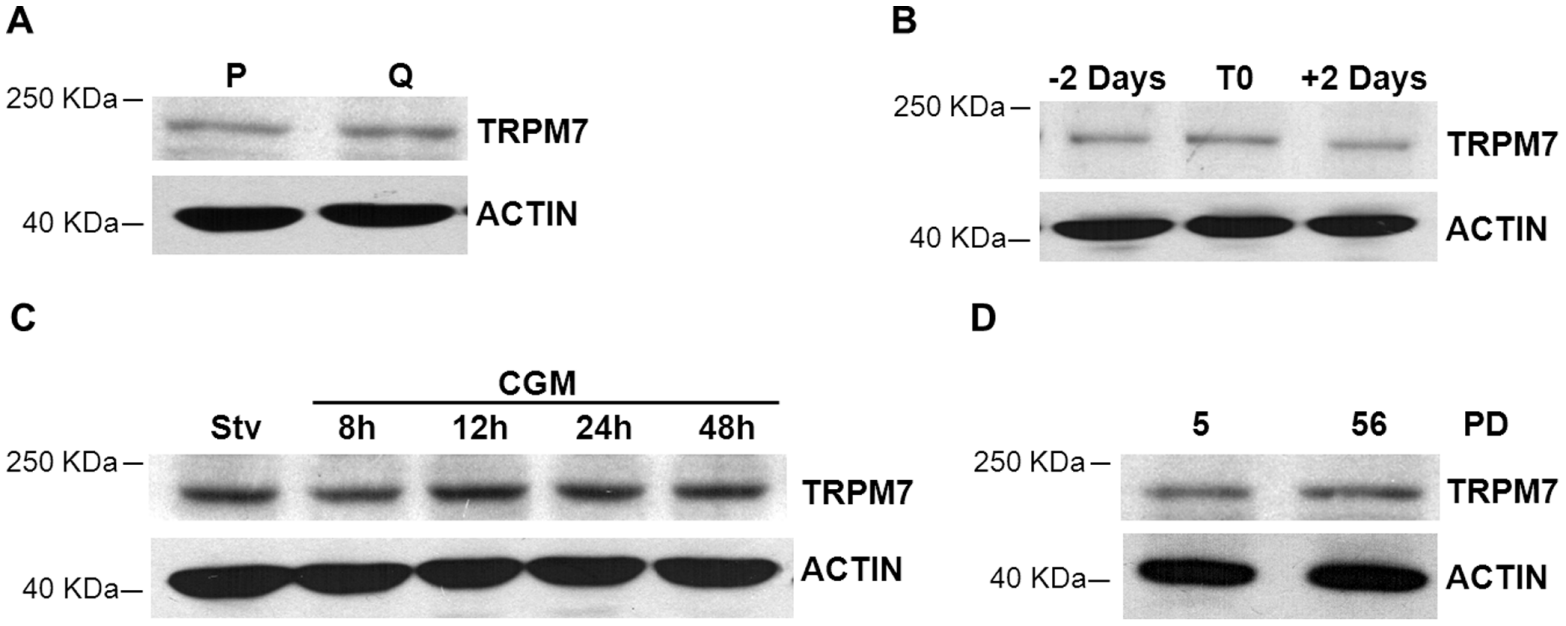
TRPM7 amounts in quiescent, proliferating and senescent HUVEC. (A) HUVEC were either cultured in complete growth medium (proliferating cells, P) or in starvation medium (quiescent cells, Q) for 2 days. (B) HUVEC were lyzed 2 days before (−2, preconfluent), 2 days after reaching confluence (+2, post-confluent) and on the day (T0) they reached confluence. (C) Subconfluent HUVEC were starved as described for 48 h. Then the cells were exposed for different times to complete growth medium (CGM) to induce the re-entry in the cell cycle. (D) High and low PD HUVEC were analyzed for TRPM7 levels. Cell extracts were utilized for Western blot using anti-TRPM7 antibodies. Actin was used to show that equal amounts of proteins were loaded per lane.

10 h after seeding the cells, half of the samples were growth arrested by exposure to a starvation medium. After 48 h, the cells were lyzed and Western blot performed. No significant modulation of the total amounts of TRPM7 was observed in cells rendered quiescent by starvation vs proliferating cells (Fig. 1A). Similarly, we compared the levels of TRPM7 in sparse vs confluent HUVEC immediately or 2 days after reaching confluence. We found no difference in the total amounts of TRPM7 (Fig. 1B). To further explore the relation between the levels of TRPM7 and proliferation, we starved HUVEC for 48 h before re-exposing the cells to their complete growth medium. Under these conditions, the entry into the S phase occurs after 24 h from the re-addition of growth medium as detected by ^3^H-Thymidine incorporation assay [15]. Upon re-entry in the cell cycle, no differences of the total amounts of TRPM7 were detected (Fig. 1C).

Last, since cellular senescence is linked to growth arrest, it is noteworthy that the amounts of TRPM7 did not change in HUVEC rendered senescent by serial *in vitro* passage when compared to early passage, young cells (Fig. 1D). All together, these results indicate that the levels of TRPM7 are not modulated by endothelial quiescence or senescence.

### Modulation of TRPM7 Expression by Different Concentrations of Mg

**Figure 2:**
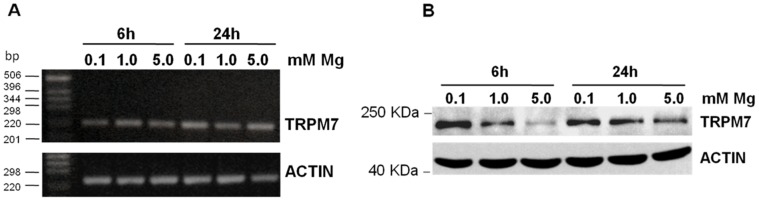
TRPM7 expression in endothelial cells cultured in different concentrations of Mg. (A) RT-PCR was performed on RNA extracted from HUVEC exposed to different concentrations of Mg for 6 and 24 h using primers designed on *TRPM7* sequence. (B) HUVEC were cultured in media containing different concentrations of Mg for 6 and 24 h. After lysis cell extracts were utilized for Western blot using anti-TRPM7 antibodies. In A and B actin was used as a control of loading.

We next investigated whether TRPM7 was modulated by different extracellular concentrations of Mg. HUVEC were cultured in 0.1, 1.0 or 5.0 mM Mg for various times. While *TRPM7* transcript did not change as detected by semiquantitative RT-PCR (Fig. 2A), by Western blot we observed that the total amounts of TRPM7 were increased by approximately 2 fold in HUVEC exposed for 6 h to 0.1 mM Mg and decreased thereafter. On the contrary, culture of HUVEC in 5.0 mM Mg reduced the total amounts of TRPM7 (2 fold) (Fig. 2B).

**Figure 3:**
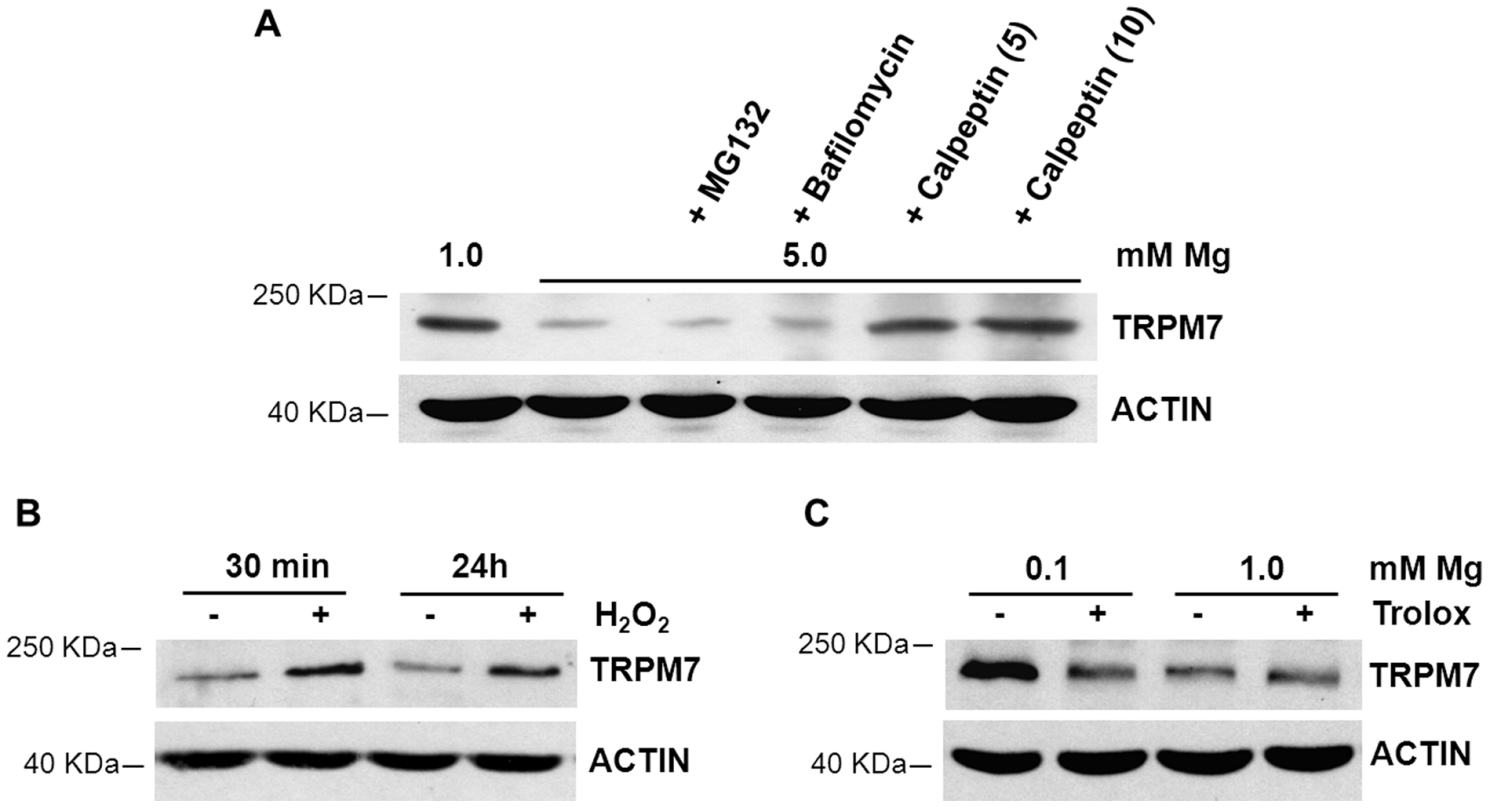
TRPM7 amounts in HUVEC treated with protease inhibitors, H_2_O_2_ or the antioxidant trolox. (A) Effects of bafilomycin, MG132 and calpeptin on TRPM7 total amounts. HUVEC were cultured in 5.0 mM Mg in the presence of MG132 (5 µM), bafilomycin (100 nM) or calpeptin (5 and 10 µg/ml) for 24 h. (B) Effects of H_2_O_2_ (100 µM) on TRPM7 total amounts in HUVEC. The cells were lysed 30 min or 24 h after adding H_2_O_2_. (C) Effects of trolox (10 µM) on TRPM7 total amounts in HUVEC. HUVEC cultured in 0.1 or 1.0 mM Mg were treated with trolox for 24 h. Western blot was performed as described above. Actin was used to show that equal amounts of proteins were loaded per lane.

To highlight the mechanisms involved in the decrease of TRPM7 by high Mg, we cultured HUVEC in 5.0 mM Mg for 24 h in the presence of MG132 (5 µM), an inhibitor of the proteasome, bafilomycin (100 nM), which inhibits lysosomal activity, or calpeptin (5 and 10 µg/ml), a specific m- and μ-calpain inhibitor [17]. We found that, while bafilomycin and MG132 had no significant effect, calpeptin increased the amounts of TRPM7 in HUVEC in 5.0 mM Mg (Fig. 3A).

We then tried to understand the mechanisms underlying the increase of TRPM7 levels in HUVEC cultured in low extracellular Mg. Since i) low Mg promotes oxidative stress in endothelial cells [2] and *TRPM7* expression is increased by oxidative stress in monocytes and PC12 cells [18]–[19], we first evaluated whether hydrogen peroxide modulated the amounts of TRPM7. As indicated in fig. 3B, the addition of H_2_O_2_ (100 µM), from which hydroxyl radicals are produced by the Fenton reaction, rapidly and stably increased TRPM7 levels after 30 min and up to 24 h. In addition, the antioxidant trolox (10 µM) prevented TRPM7 accumulation in HUVEC cultured in 0.1 mM Mg containing medium (Fig. 3C).

### Role of TRPM7 in HUVEC Proliferation, Migration and Nitric Oxide Synthesis

**Figure 4:**
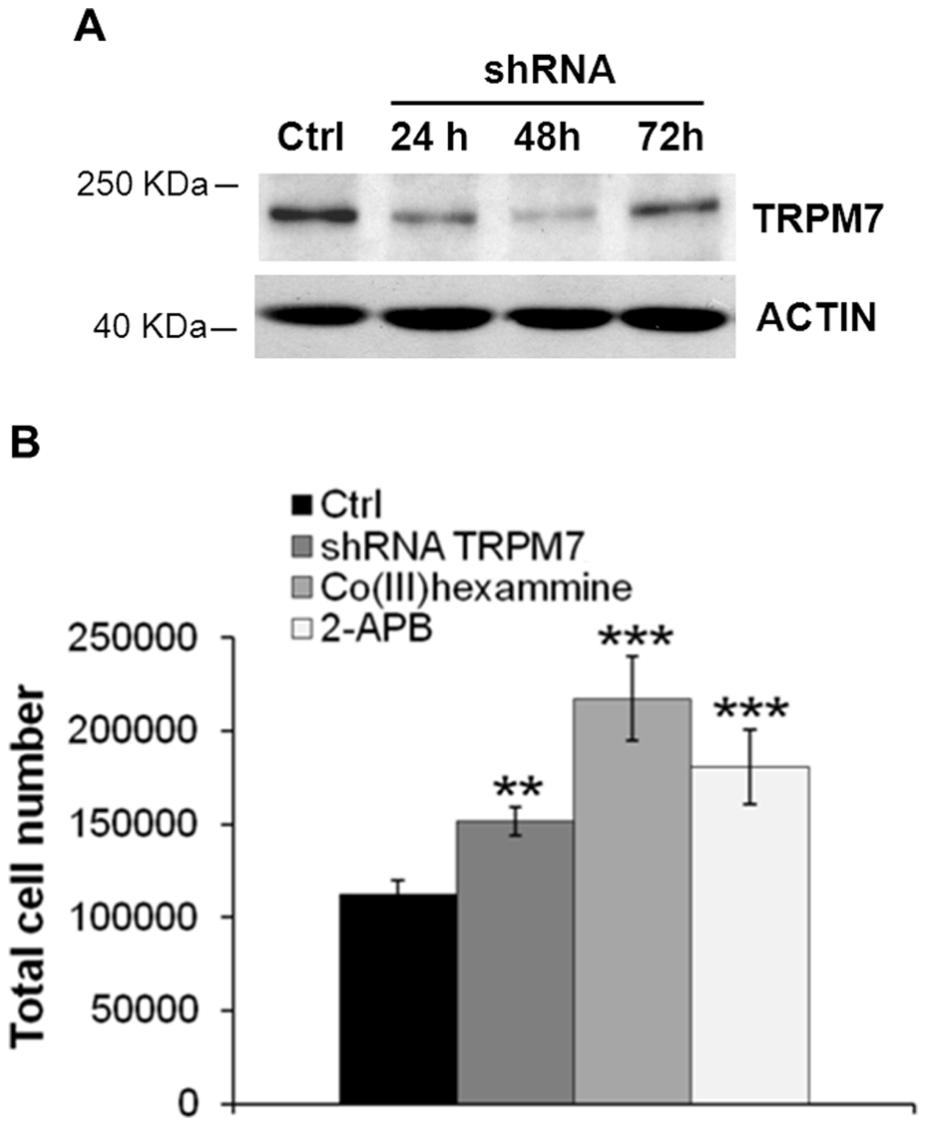
Effect of TRPM7 inhibition on HUVEC proliferation. (A) Western blot was performed on cell extracts of HUVEC transfected for 24, 48 and 72 h with shRNA against *TRPM7*. (B) HUVEC were treated with 2-APB and Co(III)hexaammine or transfected with a shRNA against *TRPM7*. After 72 h, HUVEC were harvested by digestion with trypsin, and viable cells were counted using a Burker chamber. Data refer to three separate experiments in triplicate ± standard deviation. **p<0.01, ***p<0.001.

To determine whether TRPM7 contributes to the proliferation of endothelial cells, we transiently transfected HUVEC with a specific shRNA or with a non-silencing shRNA sequence as a control. TRPM7 levels were dramatically reduced 24 and 48 h after transfection and increased thereafter (Fig. 4A). We detected a statistically significant induction of cell growth in HUVEC transiently transfected for 72 h vs their controls (Fig. 4B). We also show growth induction by 2-APB (50 µM) and Co(III)hexaammine (250 µM), two commonly used non-specific blockers of TRPM7 activity [20]–[21] (Fig. 4B). We conclude that both pharmacological and genetic inhibition of TRPM7 stimulate endothelial growth.

**Figure 5:**
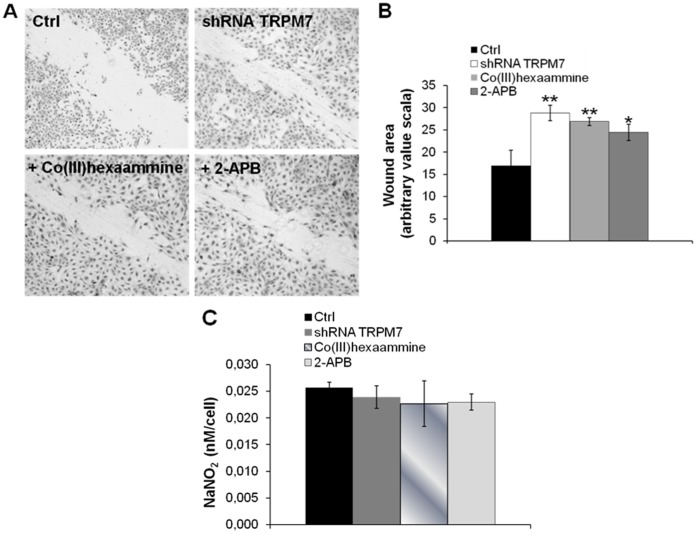
Effect of TRPM7 inhibition on HUVEC migration and NO production. HUVEC were treated with 2-APB and Co(III)hexaammine or transfected with a shRNA against *TRPM7* for 24 h. (A) The cells were wounded and migration was allowed for 10 h. Microphotographs show the result of a representative assay (20×magnification). (B) The wound area was calculated by ImageJ software and expressed using an arbitrary value scale to quantify the results. The values are expressed as the mean of 3 different experiments ± standard deviation. *p<0.05, **p<0.01. (C) NOS activity was measured by using the Griess method.

We then evaluated cell migration by wound assay and found that silencing *TRPM7* as well as treatment with 2-APB (50 µM) and Co(III)hexaammine (250 µM) induced HUVEC migration (Fig. 5A and B).

Nitric oxide (NO) is a vasoprotective factor with anti-atherogenic properties [22], also implicated in endothelial proliferation [16]. We found no alterations of NO production after 24 h exposure to 2-APB (50 µM), Co(III)-hexaammine (250 µM) or after 24 h *TRPM7* silencing (Fig. 5C).

## Discussion

TRPM7 is an important regulator of Mg homeostasis and adequate concentrations of Mg contribute to maintain normal endothelial functions [9], [13]. Since i) the functional and structural integrity of the endothelium is critical for vascular homeostasis and ii) TRPM7 is emerging as an important modulator of vascular functions, we first investigated the modulation of the expression of TRPM7 in HUVEC, and then its function by analyzing cell proliferation, migration and NO synthesis in HUVEC exposed to pharmacological inhibitors of TRPM7 or genetically engineered to silence it.

In general, the expression of ion channels is rather stable, being conductivity regulated by gating mechanisms linked to signalling cascades. Indeed, in vascular smooth muscle cells exposed to fluid flow, TRPM7 expression does not change and the increase in TRPM7 current is due to its translocation to the plasma membrane [12]. However, factors influencing TRPM7 expression have been described, and this might be due to the fact that TRPM7 is not only a ion channel but also a protein kinase. In smooth muscle cells angiotensin II and aldosterone modulate TRPM7 abundance and influence TRPM7 dependent Mg transport [23]. In PC12 cells, LPS and hydrogen peroxide strongly increase the expression of TRPM7 [19]. Accordingly, *TRPM7* expression is enhanced by oxidative stress in monocytes [18]. In addition, TRPM7 is overexpressed in breast and pancreatic adenocarcinomas [24], [25]. Because TRPM7 is involved in the regulation of cell growth and senescence [26], we studied whether these events impact on TRPM7 levels in HUVEC. We did not observe any significant modulation of TRPM7 in proliferating, quiescent and senescent HUVEC, but we found that the levels of TRPM7 depend upon extracellular concentrations of Mg. In particular, low extracellular Mg increases while high extracellular Mg decreases TRPM7 in the absence of any modulation of its transcript, suggesting the possibility of a post-transcriptional regulation. We demonstrate that the accumulation of TRPM7 in HUVEC cultured in 0.1 mM Mg is mediated by free radicals. Indeed, trolox, a water soluble analogue of α-tocopherol which protects against lipoperoxidation, prevents low Mg-induced upregulation of TRPM7. The role of oxidants in inducing TRPM7 in HUVEC is further demonstrated by the evidence that hydrogen peroxide increases TRPM7 levels, in agreement with the results obtained in PC12 [19]. Because low Mg promotes endothelial dysfunction partly through the action of free radicals, we propose TRPM7 overexpression as a potential marker of macrovascular endothelial dysfunction. Notably, increased TRPM7 expression was reported in aortas from MgL mice, a model in which endothelial dysfunction is detected [4]. TRPM7 is increased also in human osteoblast-like cells grown in low Mg but the mechanisms involved have not been revealed [27]. In HUVEC under Mg deficiency, we hypothesize that the increase of TRPM7 levels might ensure an adequate supply of the cation for cell activities. Indeed, intracellular Mg remains unvaried in HUVEC grown in 0.1 and 1.0 mM Mg [1]. Intriguingly, we have recently shown decreased amounts of TRPM7 in microvascular endothelial cells cultured in low Mg. This event is linked to growth inhibition [10]. The different regulation of TRPM7 between HUVEC and HMEC by low Mg can be ascribed to the fact that low Mg does not induce oxidative stress in HMEC. Accordingly, antioxidants do not prevent low Mg effects on these cells [10]. On the other hand, the differences observed in the behaviour of HMEC and HUVEC are not surprising, since endothelial cells from different vascular beds are heterogeneous, and HUVEC and HMEC present many differences in basal gene expression profiles [28].

In HUVEC cultured in high Mg, the levels of TRPM7 decrease. We hypothesize that this downregulation might protect the cells against Mg overload which would negatively influence TRPM7 channel activity. Indeed, as intracellular Mg increases, TRPM7 activity declines. In addition, too much intracellular Mg would generate imbalances of ion concentrations, in particular interfering with calcium homeostasis [29]. We found that the reduction of TRPM7 levels in HUVEC exposed to high Mg depends upon the activation of calpains. Calpains are members of a large family of intracellular calcium-dependent cysteine proteases which are involved in a large number of physiological and pathological phenomena [30]. The best characterized calpains, µ-calpain and m-calpain, are two typical and ubiquitous isoforms [30]. While m-calpain is activated by millimolar calcium (0.1–1 mM), micromolar concentrations (5–50 µM) of calcium are necessary to activate µ-calpain. In addition, m-calpain can be activated through direct phosphorylation by ERK [31]. In our experimental model, however, we were unable to detect any relevant modulation of ERK phosphorylation [10]. More studies are required to understand the mechanisms leading to calpain activation in HUVEC cultured in 5.0 mM Mg. It is noteworthy that TRPM7 has been shown to be a potent regulator of m-calpain and to colocalize with the enzyme at peripheral vinculin-containing adhesion complexes in the HEK293 cell line [32]–[33]. We hypothesize the existence of a complex interplay between calpain and TRPM7 where TRPM7 is not only a regulator of calpain activity but also a target of the enzyme.

Last issue to consider is the role of TRPM7 in regulating HUVEC function.

An adequate production of NO is a marker of endothelial function to the point that in clinical practise the examination of vasodilatation in response to stimuli that release NO is routinely employed to assess endothelial function [1], [22]. We did not find any change in NO production both after silencing *TRPM7* and inhibiting its activity with 2-APB or Co(III)hexaammine. The fact that both pharmacological and genetic inhibition of TRPM7 have no effect on NO production is relevant, since our results with shRNA against *TRPM7* are in disagreement with a previous report showing that silencing *TRPM7* stimulates NO production in an ERK pathway dependent fashion [9]. Possible reasons for this discrepancy are the different conditions utilized to culture HUVEC and silence them as well as the different methods used to measure NO.

Both genetic and pharmacological inhibition of TRPM7 induce HUVEC proliferation. To our knowledge, HUVEC are unique in responding with an increase of cell growth to *TRPM7* silencing. In particular, it is worth to note that human microvascular endothelial cells are arrested in the G1 phase of the cell cycle upon silencing *TRPM7* [10], a result that further underscores that TRPM7 serves different functions in endothelial cells of different origin. Again, while *TRPM7* silencing significantly impairs HMEC motility [10], TRPM7 inhibition stimulates HUVEC to migrate. We hypothesize that HUVEC and microvascular endothelial cells might express different Mg transporters. In particular, it is feasible to propose that other Mg channels vicariate TRPM7 functions in HUVEC. Studies are in progress to address this issue.

The evidence that TRPM7 inhibition impacts on endothelial cell migration and proliferation might have pathophysiologic significance. Indeed, low levels of TRPM7 might facilitate the re-endothelization of vascular injuries, thus preventing excessive subintimal proliferation of smooth muscle cells and reducing the risk of vascular complications. If this theory proves true, maintaining low levels of TRPM7 might contribute to vascular integrity and, in case of damage, to vascular repair.
